# The association between cerebral developmental venous anomaly and concomitant cavernous malformation: an observational study using magnetic resonance imaging

**DOI:** 10.1186/1471-2377-14-50

**Published:** 2014-03-15

**Authors:** Guolu Meng, Chuanfeng Bai, Tengfei Yu, Zhen Wu, Xing Liu, Junting Zhang, Jizong zhao

**Affiliations:** 1 Department of Neurosurgery, Beijing Tiantan Hospital, Capital Medical University, 6 Tiantan Xili, Chongwen District, Beijing 100050, People’s Republic of China

**Keywords:** Developmental venous anomaly (DVA), Cavernous malformation (CM), Magnetic resonance imaging (MRI)

## Abstract

**Background:**

Some studies reported that cerebral developmental venous anomaly (DVA) is often concurrent with cavernous malformation (CM). But there is lack of statistical evidence and study of bulk cases. The factors associated with concurrency are still unknown. The purpose of this study was to determine the prevalence of concomitant DVA and CM using observational data on Chinese patients and analyze the factors associated with the concurrency.

**Methods:**

The records of all cranial magnetic resonance imaging (MRI) performed between January 2001 and December 2012 in Beijing Tiantan Hospital were reviewed retrospectively. The DVA and CM cases were selected according to imaging reports that met diagnostic criteria. Statistical analysis was performed using the Pearson chi-square statistic for binary variables and multivariable logistic regression analysis for predictors associated with the concurrent CM.

**Results:**

We reviewed a total of 165,230 cranial MR images performed during the previous 12 year period, and identified 1,839 cases that met DVA radiographic criteria. There were 205 patients who presented concomitant CM among the 1,839 DVAs. The CM prevalence in DVA cases (11.1%) was significantly higher than that in the non-DVA cases (2.3%) (P<0.01). In the multivariate analysis, we found that DVAs with three or more medullary veins in the same MRI section (adjusted OR = 2.37, 95% CI: 1.73-3.24), infratentorial DVAs (adjusted OR = 1.71, 95% CI: 1.26-2.33) and multiple DVAs (adjusted OR = 2.08, 95% CI: 1.04-4.16) have a higher likelihood of being concomitant with CM.

**Conclusions:**

CM are prone to coexisting with DVA. There is a higher chance of concurrent CM with DVA when the DVA has three or more medullary veins in the same MRI scanning section, when the DVA is infratentorial, and when there are multiple DVAs. When diagnosing DVA cases, physicians should be alerted to the possibility of concurrent CM.

## Background

Cerebral vascular malformation (CVM) is a class of important central nervous system diseases. According to Russell and Rubinstein’s standard, there are four major types of vascular malformations of the central nervous system: developmental venous anomaly (DVA), cavernous malformation (CM), arteriovenous malformation, and capillary telangiectasia [1]. DVA and CM are the two most common diseases among CVM. DVA makes up 42 – 63% of all CVM [2]. CM accounts for 5-13% of all CVM [3].

DVA is a congenital abnormality of venous drainage [4] composed of radially arranged venous complexes converging into a centrally located venous trunk, which drains the normal brain parenchyma [5]. DVAs have a benign clinical course [6], and the reported incidence is 0.05-3% based on enhanced imaging and autopsy in the general population [6-10]. CM is a vascular lesion that lacks the features of arteries or veins [11,12]. CMs occur in about 0.4% of autopsies. The pathogenesis of CM includes pathological factors as well as genetic mutation [13].

Most DVAs and CMs are asymptomatic and are discovered incidentally through neuroimaging. MRI is an effective imaging tool for detecting DVA and CM [7,14] because both DVA and CM have respective characteristic imaging [11]. Other methods such as digital subtraction angiography can be used for diagnosis, but with the wide application of MRI, and noninvasive features, MRI diagnosis is often a preferable alternative. For the purposes of identifying both DVA and CM, MRI is also better suited since most CMs cannot be diagnosed using angiography.

Past studies found that DVA is often concurrent with CM. CM is reported in the literature to have an association with DVA at a rate of 2%–33% [4,12,15-18]. The presence of CM with or without DVA will influence treatment [19]. There are several documented reasons for the concurrency. There is increased systemic or local venous pressure in the DVA [20]. Increased venous pressure may lead to recurrent petechial congestional hemorrhage [21,22], or may produce ischemia which stimulates the growth of new vessels [23]. These new vessels are fragile and susceptible to bleeding, and repeated hemorrhaging may subsequently form a CM [7,21].

In this study, we aimed to determine the prevalence of concurrent DVA and CM using observational data on Chinese patients and to analyze the factors associated with concurrency.

## Methods

### Study population and data collection

From January 1, 2001 to December 31, 2012, a total of 165,230 patients received MRI screening at Beijing Tiantan Hospital. Data were collected from the patients’ MRI registration system and MRI reports. For patients who underwent multiple MRI screenings over the study period, only the most recent screening results were included in this analysis. Of these patients, 1,839 patients were diagnosed with DVA.

According to the standard procedures of Beijing Tiantan Hospital, all MRI images were read by two radiologists, including at least one senior-level radiologist. The final diagnosis was approved by both radiologists. In rare cases when the radiologists’ diagnosis was inconclusive, the researcher examined the original MRI images and assigned a classification to the case.

### MRI screening

During the study period,the following MRI machines used were: GE Signa 1.5 T, GE Signa 3.0 T, Toshiba Visa1.5 T, and Siemens Magnetom Trio 3.0 T superconducting magnetic resonance imager. The contrast agent was Gadopentetate dimeglumine (Gd—DTPA).

### Diagnostic criteria using MRI

DVA diagnostic criteria included presence of lesions in the white matter, typical stellate or linear vascular lesions converging into a collecting vein and draining into the dural, sinus, or deep veins, and an umbrella or caput medusa-like appearance especially on an enhanced image [7]. If three or more medullary veins were visible simultaneously in at least one section of MRI, and presented in “spoke wheel” or caput medusae configurations [24], we classified the DVA in a “≥3 medullary veins group”. When there were fewer than three medullary veins in any single section of MRI, we classified the DVA in a “<3 medullary veins group”.

CM diagnostic criteria included presence of lesions with reticulated mixed signal blood-containing locules with the classic heterogeneous “popcorn” appearance on both T1 and T2-weighted images, a rim of haemosiderin in the surrounding brain parenchyma; and minimal or no enhancement on the T1 image [7,9]. Their appearance on a MRI will depend on the degree of the hemorrhage, with T2-weighted images being the most sensitive sequence.

Hemorrhages were not classified as CMs if the hemorrhage lesions were only acute or subacute hematomas dominated by intracellular methaemoglobin, and therefore, appeared with a homogeneous signal on MRI images; and if there were only tiny, punctate foci of hypo intensity on both T1 and T2-weighted sequences, with no heterogeneous signal. On imaging, when CM diagnosis was made, other causes of a single hemorrhagic lesion, such as arteriovenous malformation, bland intraparenchymal hemorrhage, hemorrhagic infection, and neoplasm had to be excluded [18].

### Statistical analyses

We compared different groups using the Pearson chi-square statistic for categorical variables. We also performed multivariable logistic regression analysis to find the factors associated with concurrent CM. Independent variables included age, gender, location of DVA, largest number of medullary veins (≥3 or <3 ) in the same MRI section, and quantity of DVAs. For the age variable, age groups were formed by 20 year intervals. For the location variable, patients were divided into two groups, supratentorial and infratentorial. All confidence intervals reported were 95%, and all p-values were two-sided. P-values less than 0.05 were considered statistically significant. All statistical analyses were performed using SAS software (Version 9.1.3, SAS Institute Inc., Cary, NC, USA).

### Ethics

The study was approved by the Committee on Human Research at Beijing Tiantan Hospital.

## Results

In total, 165,230 patients received MRI screening at Beijing Tiantan Hospital from January 1, 2001 to December 31, 2012. Of these patients, 1,839 (1.11%) were diagnosed with DVA and 3,856 (2.33%) were diagnosed with CM. Among the 1,839 DVA cases,a subgroup of 205 (11.15%) cases presented with concomitant CM. In the 163,391 non-DVA cases, there were 3,651 cases of CM, and the prevalence was 2.23%. The CM prevalence in DVA cases (11.15%) was significantly higher than in the non-DVA cases (P<0.01).

Among the 1,839 DVA cases, 940 were male (51.11%) and 899 were female. The average age was 40.40 years (range: 0.25-87) with a standard deviation of 16.01 years. We detected 1,782 (96.90%) cases with a single DVA, and 57 (3.10%) cases with multiple DVA. Supratentorial and infratentorial DVA cases numbered 1,319 (71.72%) and 520 (28.28%) respectively. We found 388 (21.10%) DVA cases with three or more medullary veins visible simultaneously in at least one section of MRI, and 1,451 (78.90%) DVA cases with fewer than three medullary veins in any single MRI section.

Besides 205 concurrent CMs, there were 174 (9.46%) cases of concomitant hemorrhage or hematoma with DVAs, but the hemorrhage did not meet our diagnostic criteria for CM in MRI, and thus were not classified as CM.

Among the 205 DVA associated with CM cases,169 (82.44%) had CMs adjacent to DVAs and located at the distal radicles of DVAs. There were 188 (91.71%) cases with a single CM lesion, and 17 cases with multiple CMs.

In the multivariate analysis, concomitant CM was associated with three variables. Concomitant CM was almost twice as likely to occur when DVA was in an infratentorial location (adjusted OR = 1.71, 95% CI: 1.26-2.33, P = 0.00). Additionally, CM was 2.37 times more likely to occur with DVA if there were three or more medullary veins visible in one MRI section (95% CI: 1.73-3.24, P = 0.00). Though multiple DVAs were not significantly associated with concomitant CM in the univariate analysis (P = 0.051), the likelihood of concomitant CM was 2.08 times more (95% CI: 1.04-4.16, P = 0.038) holding all other variables constant. Finally, though gender was significantly associated with concomitant CM in the univariate analysis, there was no association in the multivariate analysis (Tables 1 and 2).

**Table 1:** Prevalence of CM among DVA and non-DVA cases by MRI imaging between 2001–2012 at Beijing Tiantan hospital

**DVA type**	**CM**	**Non- CM**	**Total**	**CM prevalence**
**DVA**	205	1634	1839	11.15%
**Non-DVA**	3651	159740	163391	2.23%
**Total**	3856	161374	165230	2.33%

*X*^2^ = 617.75, P<0.01.

**Table 2:** Clinical and imaging predictors of concomitant CM among DVA cases

**Characteristics**	**Total N**	**CM N (%)**	**Non CM N (%)**	**P-value**	**Unadjusted OR**^**† **^**(95% CI**^**§**^**)**	**P value**	**Adjusted OR**^**‡ **^**(95% CI**^**§**^**)**	**P-value**
**Overall**	**N = 1839**	**N = 205**	**N = 1634**					
Age	**.**					.		
Median (IQR), years	41.5(29–52)	39(27–49)	42(29–52)	0.0242				
				0.2299				
<20	210	25(11.9%)	185(88.1%)		1.0	.	1.0	.
20- 39	617	80(13%)	537(87%)		1.10(0.68–1.78)	0.6899	1.06(0.65–1.72)	0.2037
40-59	801	82(10.2%)	719(89.8%)		0.84(0.52–1.36)	0.4848	0.85(0.53–1.39)	0.6832
> = 60	211	18(8.5%)	193(91.5%)		0.69(0.36–1.31)	0.2555	0.72(0.38–1.38)	0.2760
Gender	.			0.0241		.		
Female	899	85(9.5%)	814(90.5%)		1.0	.	1.0	.
Male	940	120(12.8%)	820(87.2%)		1.40(1.04–1.88)	0.0246	1.30(0.97–1.76)	0.0839
DVA Location	.			<.0001		.		
Supratentorial	1319	121(9.2%)	1198(90.8%)		1.0	.	1.0	.
Infratentorial	520	84(16.2%)	436(83.8%)		1.91(1.41–2.57)	0.0000	1.71(1.26–2.33)	0.0006
DVA imaging	.			<.0001		.		
Medullary veins ≥ 3	1451	128(8.8%)	1323(91.2%)		1.0	.	1.0	.
Medullary veins<3	388	77(19.8%)	311(80.2%)		2.56(1.88–3.48)	0.0000	2.37(1.73–3.24)	0.0000
Quantity of DVAs	.			0.0470		.		
Single	1782	194(10.9%)	1588(89.1%)		1.0	.	1.0	.
Multiple	57	11(19.3%)	46(80.7%)		1.96(1.00–3.84)	0.0510	2.08(1.04–4.16)	0.0384

OR: odds ratio, CI: confidence interval, IQR: inter-quartile range.

^†^Unadjusted odds ratios and p-values generated via univariate analysis.

^‡^Adjusted odds ratios and p-values resulted from multivariate analysis using a logistic regression model. All variables in univariate analysis were included in the multivariable model.

^§^All CI presented are 95% CI and all p-values presented are two-sided.

## Discussion

Previous studies indicate that DVA and CM are the two most common central nervous system diseases among vascular malformations, and are frequently found coexisting [24]. However, most of these studies were based on small numbers of cases outside of China. The prevalence and imaging features of coexisting DVA and CM in the Chinese population needs to be clarified. Using MRI detection, our study indicates that morbidity of CM in DVA cases is significantly higher than in the non-DVA population. We also found a higher likelihood of concomitant CM when the DVA had three or more medullary veins in the same MRI section, when the DVA was infratentorial, and when multiple DVAs were present.

We found a 1.11% prevalence of DVA and an even higher prevalence of CM at 2.33%. In the literature, DVAs are the most common intracranial vascular malformation. There are two possible explanations for this discrepancy. First, our study sample is not representative of the general population. Beijing Tiantan Hospital is one of the largest neurosurgery centers in China, and many patients are referred to this hospital for CM diagnosis and treatment. In comparison, more DVAs are asymptomatic than CMs. Second, all of our patients were from China. It is possible that CM is the most common intracranial vascular malformation among Chinese people. Among the 1,839 patients with DVA, 205 also had CM. The prevalence of concurrent CM in DVA cases was 11.15%, which is significantly higher than CM non-DVA cases (2.23%, P<0.01). The coexistence of DVA and CM has been recognized by some researchers [11,17,25,26]. In DVA cases, the most common coexisting vascular anomaly was CM [24]. CM are reported in the literature to have an association with DVA at a rate of 2%–33% [4,12,15-18]. In our study the rate was 11.15% (205/1839). In most studies, once a hemorrhage or hematoma was detected in DVA cases, the diagnosis of CM would be made [2,4,21,27]. In our study, there were also 174 cases of concomitant hemorrhage or hematoma with DVA. However, we did not diagnose these as CM because the hemorrhage did not meet our diagnostic criteria for MRI-detected CM. If we classified patients who had any hemorrhage as CM cases, the concurrent rate would be higher, reaching 20.61%.

**Figure 1:**
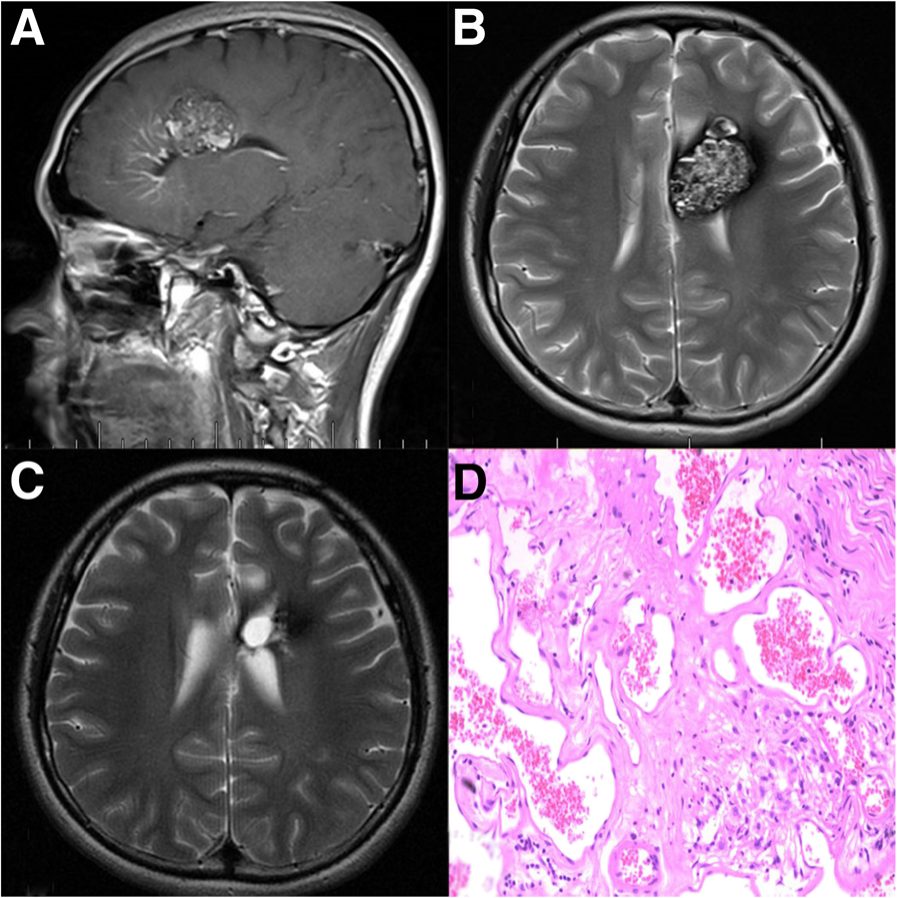
**Supratentorial DVA and Concomitant CM. A**: Enhanced MRI image showing the DVA and the CM which locates at the distal radicles of the DVA. **B**: CM shows classic heterogeneous “popcorn” appearance on T2-weighted image. **C**: Post-operation image showing that the CM disappeared. **D**: Pathological picture of the CM showing the sinusoidal vascular channels.

**Figure 2:**
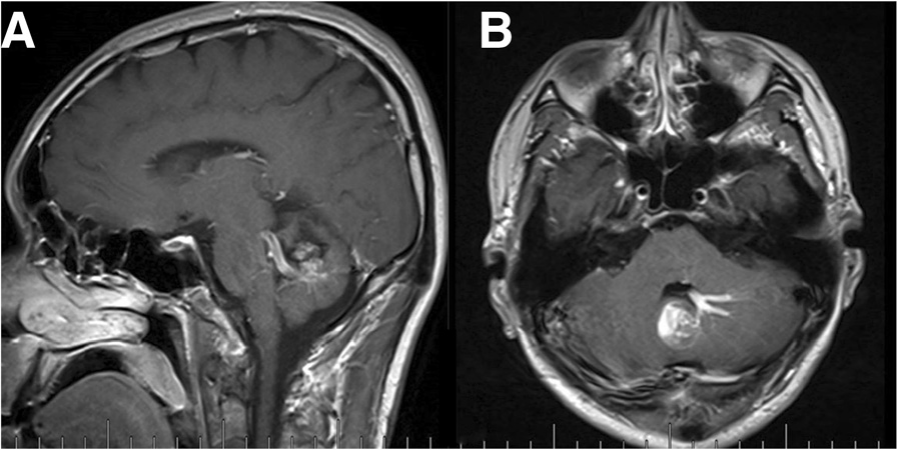
**Subtentorial DVA and Concomitant CM. A** and **B**. Enhanced MRI images showing irregular “caput medusae” in the left cerebellar hemisphere and pons, and the CM locates at the distal radicles of the DVA.

The close relationship between CM and DVA may suggest that formation of CM is caused by DVA. Elevated venous pressure in DVA often leads to tiny recurrent hemorrhages [21,22]. Self-limited recurrent hemorrhages [28] may subsequently form a CM [7,21]. Among the 205 coexisting CM cases in DVAs, 169 cases (82.44%) had CMs adjacent to the DVAs and located at the distal radicles of DVAs. This phenomenon indicates a close relationship between CM and DVA. Thus, follow-up of DVA cases with hemorrhage is warranted because repeated hemorrhage may likely lead to CM formation (Figures 1 and 2).

Our study found a higher likelihood of concurrent CM in DVAs with three or more medullary veins occurring in one MRI section, in infratentorial DVAs, and when multiple DVAs were present. The presence of several medullary veins may lead to increased venous pressure and a greater chance of bleeding. While it is difficult to measure the pressure of DVA, CM pressure has been detected during surgery and reported to be markedly higher than cortical venous pressure [29].

When a patient has a DVA that is infratentorial and has three or more medullary veins occurring in one MRI section, there is a 16% chance of concomitant CM, which is significantly higher than that of supratentorial DVA. Studies suggest that DVAs in the posterior fossa are more likely to hemorrhage than their supratentorial counterparts [30].

Multiple DVAs were highly associated with concurrent CMs. No other study has reported this finding. Due to a low proportion of multiple DVAs, this may be a random event. However there is a possibility that multiple DVAs foster a greater opportunity for the development of CM lesions.

The age at presentation of CM is usually between 40 to 60 years [28]. In the literature, the coexistence of CM and DVA is more common in adults than children [31]. We found no statistical significance between the concurrent rates of CM in our four age groups. Moreover, our analysis did not show that gender was related to concomitant CM.

MRI is an extremely sensitive approach for detecting CM [7,14]. Radiologists have strongly recommended diagnosing CM based on characteristic MRI features, thus avoiding additional invasive procedures like digital subtraction angiography and surgical biopsy. The most common appearance of CM is known as a “popcorn” lesion, which involves a nucleus with a heterogeneous signal in T1- and T2-weighted images surrounded by a complete hemosiderin ring with lower signal intensity in T2-weighted sequences [3]. The appearance of CM varies based on the degree of the hemorrhage. CMs have a propensity to grow over time [28]. The typical presence of CM coexisting with DVA may originate from repeated small hemorrhages [17,28,31-33].

Most CMs are solitary lesions, whereas 10–30% are multiple form [12,14,28,34]. Most cases with a single lesion are sporadic form. This difference in the imaging features of familial and sporadic CM suggests that the two have different developmental mechanisms [12]. Familial cases show more multiplicity and often have a dominant pattern of inheritance due to a gene mutation localizable in the CCM1, CCM2, or CCM3 gene loci [6,35,36]. Familial CMs have different pathogenetic mechanisms than sporadic CMs [25]. Among the 205 concurrent CM cases, 188 (91.71%) cases had a single CM lesion, so it may be inferred that most of our cases are sporadic CMs rather than genetic familial cases. Sporadic CM is highly associated with DVA, because DVA is involved in the formation and development of the sporadic CM.

### Limitations

The results of this study should be interpreted with the following limitations. First, this was a cross-sectional observational study, and data in the patients’ registration system and MRI reports were limited. However, the sample size was quite large. Thus, these results may be generalizable to the involving patients. Second, we only used the images from the MRI scan to diagnose cases. Clinical and angiographical data were not included in this study. A few DVAs are not detected on CT/MRI, but may be seen in the venous phase of angiography [21,37]. Most DVA and CM cases have a benign presentation and clinical process, and most of them are discovered accidentally. However, CM is occult in angiography and most researchers consider that MRI is sensitive to both DVAs and CMs. As a result, we only used the MRI data to study the association between DVA and concomitant CM. Finally, because cases were identified by searching the diagnosis reports in the computer image system of Beijing Tiantan Hospital, DVA and CM that were missed on initial diagnosis would have been excluded from the study. To test the extent of this problem, we reviewed an additional 100 randomly selected MRI examinations without DVA or CM diagnosis. None of these cases revealed DVA or CM findings, suggesting that the case selection method of this study was unlikely to have missed cases of DVA or CM. It is unlikely that our selection procedures significantly impacted the results.

## Conclusion

As common cerebral vascular malformations, CMs are prone to coexist with DVAs. Concurrent CM is more likely when a DVA has three or more medullary veins that are visible simultaneously in at least one MRI section, when DVAs are infratentorial, and when multiple DVAs are present. Therefore physicians should be alerted that presence of DVA increases the likelihood of present or future CM formation. For all DVA cases, follow up MRI evaluation is warranted to monitor possible CM development.

## Competing interests

The authors declare that they have no competing interests.

## Authors’ contributions

Dr. GM was responsible for the study design, statistical analysis of the data, and the drafting of this article. Dr. CB and Dr. TY were responsible for collection of the clinical and imaging data, and Dr. XL was responsible for the literature review. Dr. ZW was responsible for data interpretation, and review and revision of the draft. Dr. JTZ and Dr. JZZ were responsible for the study design, critical revision of the draft, and the final approval of the version to be published. All authors read and approved the final manuscript.

## Pre-publication history

The pre-publication history for this paper can be accessed here:

http://www.biomedcentral.com/1471-2377/14/50/prepub
